# An Evaluation of the Tensile Strength of Polyglactin Sutures After Immersion in Different Herbal Mouthwashes: An In Vitro Study

**DOI:** 10.7759/cureus.43407

**Published:** 2023-08-13

**Authors:** B.L Ojastha, M Jeevitha

**Affiliations:** 1 Dentistry, Saveetha Dental College and Hospitals, Saveetha Institute of Medical and Technical Sciences, Chennai, IND; 2 Periodontics, Saveetha Dental College and Hospitals, Saveetha Institute of Medical and Technical Sciences, Chennai, IND

**Keywords:** vicryl, seamcryl suture, seamcryl, polyglactin 910, silver nanoparticles, tensile strength, vicryl suture, herbal mouth rinse

## Abstract

Background

The process of suturing is essential to the healing of surgical wounds. Sutures on surgical wounds from fabric approximate ligament tissues, control haemorrhage, and assist the primary healing process in oral surgical procedures. The oral environment may cause the suture to lose its tensile strength, which causes tissue to open and spread infection. Different mouthwashes are recommended for effective oral hygiene maintenance postoperatively. Ideally, the use of mouthwashes should not alter the mechanical properties of suture materials. A suture material's tensile strength or ability to endure tension during knotting and long-term wound protection is measured.

Aim

The aim of the current study is to assess the tensile strength of polyglactin sutures following immersion in herbal mouthwashes.

Methods

Two commercially available synthetic braided polyglactin 910 absorbable sutures (Vicryl and Seamcryl) were chosen to assess their tensile strength following immersion in two kinds of herbal mouthwashes: nilavembu (*Andrographis paniculata)* silver nanoparticle mouthwash and clove-uni stevia mouthwash. The tensile strength of the sutures was evaluated using an ElectroPuls® E3000 (Instron, Norwood, MA, USA) universal testing machine. The data were transferred to IBM Statistical Package for Social Sciences (SPSS) software version 23.0 (IBM Corp., Armonk, NY, USA), wherein the tensile strength values of Vicryl and Seamcryl after immersion in two different mouthwashes compared with control were statistically analysed using a one-way analysis of variance (ANOVA) test.

Results

The tensile strength of Vicryl suture material was found to be higher in the clove-uni stevia mouthwash group than in the nilavembu silver nanoparticle mouthwash and control (p-value=0.000, which is statistically significant). The tensile strength of Seamcryl suture material was found to be higher in the clove-uni stevia mouthwash group than in the nilavembu silver nanoparticles mouthwash and control (p-value=0.001, which is statistically significant).

Conclusion

Nilavembu silver nanoparticle mouthwashes analysed in the present study decreased the tensile strength property of Vicryl suture material after immersion for 24 hours, whereas clove-uni stevia mouthwash was shown to increase the tensile strength of both Vicryl and Seamcryl suture materials. Therefore, the selection of suture material and the postoperative prescription of mouthwash should be considered for a better clinical outcome.

## Introduction

Suturing is a crucial step in the healing of surgical wounds. To achieve the main intention of healing, the flaps are positioned closely together using sutures for a predetermined amount of time [1]. The integrity of the tissue surrounding surgical incisions must be preserved through suturing. The sustained approximation of flap margins must be kept stable throughout time since it is a crucial component of effective wound closure [2]. This encourages thorough tissue restoration and results in a successful surgical treatment outcome. The inability to complete wound closure may cause a wound to dehisce or the healing process to take longer than expected, both of which can have detrimental functional and aesthetic repercussions. These materials are continuously subjected to mechanical forces from eating, talking, and facial expressions, as well as pH level variations and proteolytic enzymes produced by salivary bacteria [3]. The ability of the right suture material to cover wounds for the best healing with minimal to no strain is an important characteristic. Tensile strength is a factor in the longevity of suture material. The ratio of the greatest stress that a suture can sustain without breaking to the initial cross-sectional area of the specified material is known as the tensile strength of the material [4].

For optimal wound healing following dental surgery and to avoid postoperative problems, maintaining good oral hygiene is crucial. Traditional mouthwashes are frequently used to care for one's mouth, but they contain chemicals that could potentially harm sutures by weakening their tensile strength. In recent years, a rising number of people have turned to herbal mouthwashes as an alternative to traditional ones because of their possible therapeutic benefits and negligible negative effects.

Suturing tissues promotes vital healing and has a major role in the success of surgical procedures, maintaining the quality of tissues in the oral environment. Sutures are used mainly to ligate blood vessels and to approximate tissues together. Suture materials are broadly classified based on source (natural or synthetic), capacity to resorb (absorbable or non-absorbable), and fabrication type (braided or monofilament). Different suture materials have different degrees of perseverance, predictability, adaptability, and low inflammatory response, and determine the ideal suture material [5]. A delayed or compromised healing process occurs from improper approximation of flap margins. Dead space, equal stress distribution along deep suture lines, and preservation of tensile strength throughout the wound are required conditions for wound closure [5-6].

The effect of each mouthwash on the suture material varies. In contemporary mouthwashes, triclosan, chlorhexidine, and HiOra have all been extensively used. Triclosan suppresses the production of prostaglandin leukotrienes, a major regulator of inflammation, and delays plaque maturation. Plaque and bacteria are combated by chlorine. The natural potassium found in HiOra mouthwash helps to lessen tooth sensitivity. Additionally, it strengthens the gums and teeth while restoring the mineral makeup of the teeth. Today, herbal mouthwashes gravitate among the public as they are formulated with natural ingredients, which can be appealing as they are free from artificial additives and chemicals. These mouthwashes typically contain plant extracts, essential oils, and herbs known for their antimicrobial and healing properties. These mouthwashes are often milder and gentler compared to conventional mouthwashes that contain chlorhexidine and artificial sweeteners, alcohol, or harsh chemicals. This can be particularly beneficial for people with sensitive gums or those prone to oral irritations. The gentle nature of herbal mouthwashes makes them suitable for daily use without causing excessive dryness or discomfort. Herbal ingredients used in mouthwashes include tea tree oil, eucalyptus oil, peppermint oil, and thyme oil which possess natural antimicrobial properties. These ingredients can help combat bacteria, reduce plaque formation, and fight bad breath. Their natural antimicrobial action can contribute to overall oral hygiene. Herbal mouthwashes often contain essential oils with refreshing and pleasant aromas, such as peppermint, spearmint, or cinnamon. These oils can help mask bad breath, leave the mouth feeling fresh, and provide a more pleasant oral care experience compared to the strong and sometimes overpowering flavours of conventional mouthwashes.

The herbs employed for the mouthwash in this study include uni stevia and nilavembu silver nanoparticles. Nilavembu is a member of the Acanthaceae plant family with the scientific name *Andrographis paniculata*. The ethyl acetate extracts of nilavembu include alkaloids, flavonoids, phenols, tannins, and coumarin. Its phytochemical components contribute to the explanation of why it possesses therapeutic properties like cytotoxic, anti-inflammatory, antimicrobial, and antioxidant properties [7]. Since silver nanoparticles are now the best drug delivery mechanism available that successfully distributes pharmaceuticals, they can be created using the biological synthesis technique of preparation [8]. Silver nitrate is commonly employed for nanoparticle synthesis. Multipurpose uses of silver nanoparticles include their anti-inflammatory, anti-angiogenic, and anticancer properties.

Uni stevia's outstanding anti-tumour, anti-diabetic, and antioxidant capabilities are due to the existence of secondary metabolites called alkaloids, tannins, saponins, quinine, and triterpenes, which were discovered through a photochemical examination of the plant. The abundance of flavonoid and glycoside components in uni stevia is what gives rise to its anti-inflammatory benefits [9].

It is crucial to properly engage the basic biological mechanisms that underlie tissue inflammation, cell proliferation with extracellular matrix, and biological and functional remodelling in order to enhance wound healing without complications [10]. The compatibility and potential benefits or drawbacks of employing particular mouthwashes in dental practice can be assessed methodically in lab settings by measuring the mechanical properties of sutures after immersion in the mouthwash. The suture material loses tensile strength in the oral environment more quickly due to certain variables, which causes the tissues to become less adaptable and cause secondary infection.

Poor adaptation of the margins, hematoma, and deterioration of the affected region are all possible outcomes of sutures that have a lack of tensile strength throughout the healing period [11]. It is important to properly care for the sutured wound after surgery. Regular rinsing with saline, chlorhexidine mouthwash, betadine, or herbal mouthwashes is required to keep sutured wounds in the oral cavity clean [12]. By immersing the sutures in various herbal mouthwashes, their impact on the mechanical properties of the suture material, specifically their tensile strength, can be assessed. This information is crucial in guiding dental professionals in the selection of appropriate mouthwashes to minimise the risk of suture failure and promote optimal wound healing. This study aims to evaluate the effect of different herbal mouthwashes on the tensile strength of polyglactin suture materials, Seamcryl and Vicryl, which are commonly used in dental surgeries.

## Materials and methods

This in vitro study was carried out in the White Lab, a material testing facility at Saveetha Dental College and Hospitals, Chennai, India. The institute's scientific research board gave its approval to the study. Because there were no human participants in the study, no human ethical approval was needed. Two researchers (B. L. O. and M. J.) worked at the Saveetha White lab to plan and carry out the study.

Thirty commercially available synthetic braided polyglactin 910 absorbable sutures (Vicryl-15 and Seamcryl-15 samples) were studied. Inclusion criteria included 4-0, 45-cm sterilised braided-coated polyglactin 910 undyed or undamaged suture material. Exclusion criteria included synthetic polyglactin 910 sutures other than Vicryl or Seamcryl, size > or < 4-0, inadequate suture length, dyed, damaged, or unsterilized suture material. Vicryl (manufactured by Johnson & Johnson Private Limited, Mumbai, India) and Seamcryl (manufactured by Surgical Sutures Private Limited, Bangalore, India) are widely used synthetic braided polyglactin 910 absorbable suture materials, and therefore these materials were selected to evaluate their tensile strength after immersion in two types of herbal mouthwashes, which were prepared in Saveetha Blue Lab (an in-house biomaterials research lab): nilavembu silver nanoparticles mouthwash and clove-uni stevia mouthwash.

**Figure 1:**
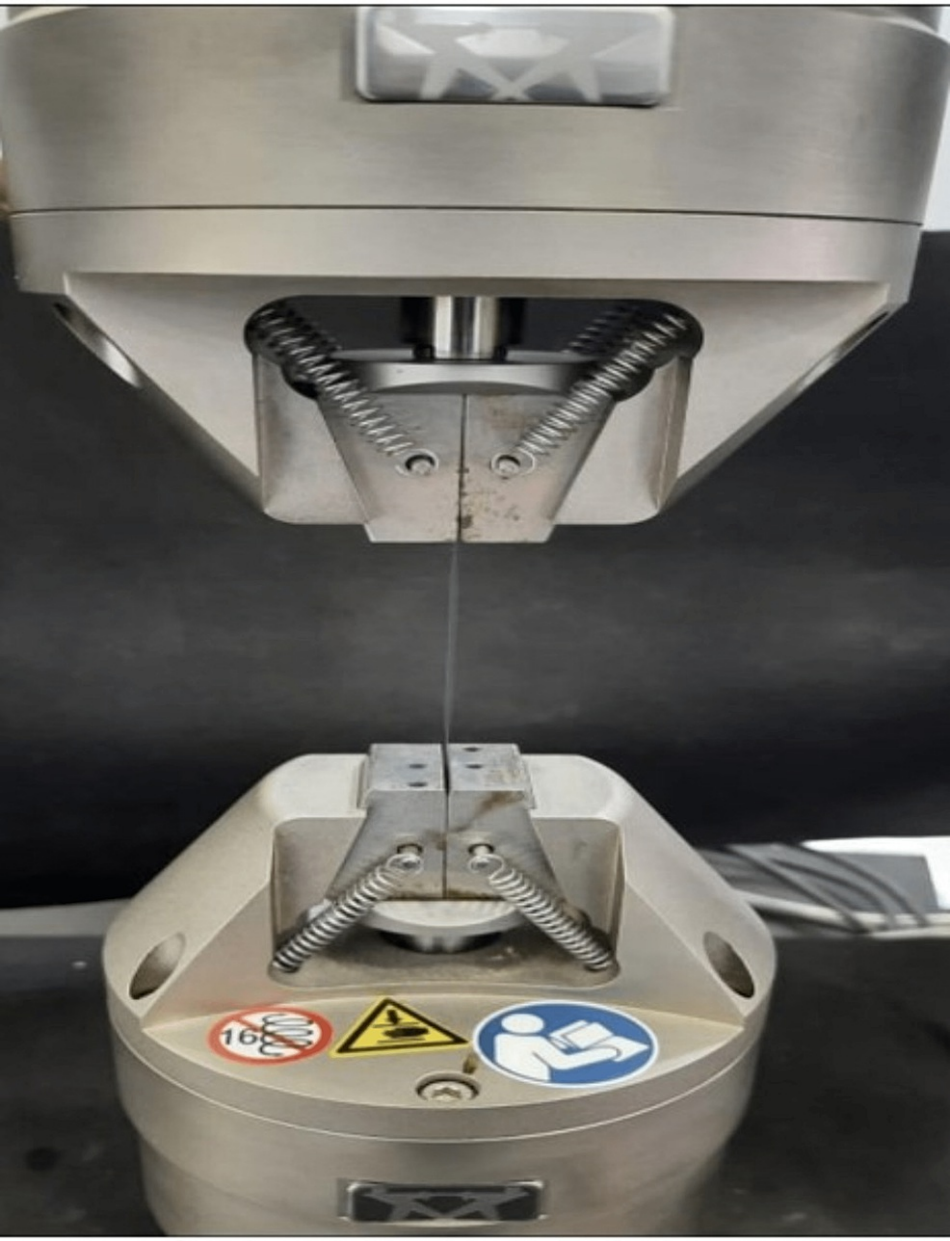
The ElectroPuls® E3000 universal testing machine measuring the tensile strength of Vicryl sutures

The present study included six groups: four test groups and two control groups. Test group 1 included Vicryl sutures immersed in nilavembu silver nanoparticles mouthwash; test group 2 included Seamcryl sutures immersed in nilavembu silver nanoparticles mouthwash; test group 3 included Vicryl sutures immersed in clove-uni stevia mouthwash; and test group 4 included Seamcryl sutures immersed in clove-uni stevia mouthwash. Control group 1 included unimmersed Vicryl sutures; control group 2 included unimmersed Seamcryl sutures. Five samples were selected for each of the test and control groups. Ten centimetres of each sample suture material were exposed to different media within in vitro settings in a controlled time frame. All the samples were tested 24 hours post-immersion in different immersion media. Control media contained air (vacuum), and test media contained mouthwashes (thermostatically controlled). The tensile strength of the test and control samples was measured using an ElectroPuls® E3000 (Instron, Norwood, MA, USA) at 10 mm/min of cross-head speed (Figure 1).

All sutures were intact at the end of their immersion period and were suitable for mechanical testing. On mechanical testing, each suture material had an evident breaking point. The data comprising the tensile strength values of all the samples were transferred to SPSS Statistics for Windows, version 23.0 (IBM SPSS Statistics for Windows, Version 23.0. Armonk, NY: IBM Corp) wherein the tensile strength values of each of the two types of absorbable suture materials after immersion in two different mouthwashes compared with control were statistically analyzed using a one-way analysis of variance (ANOVA) test. 

## Results

**Figure 2:**
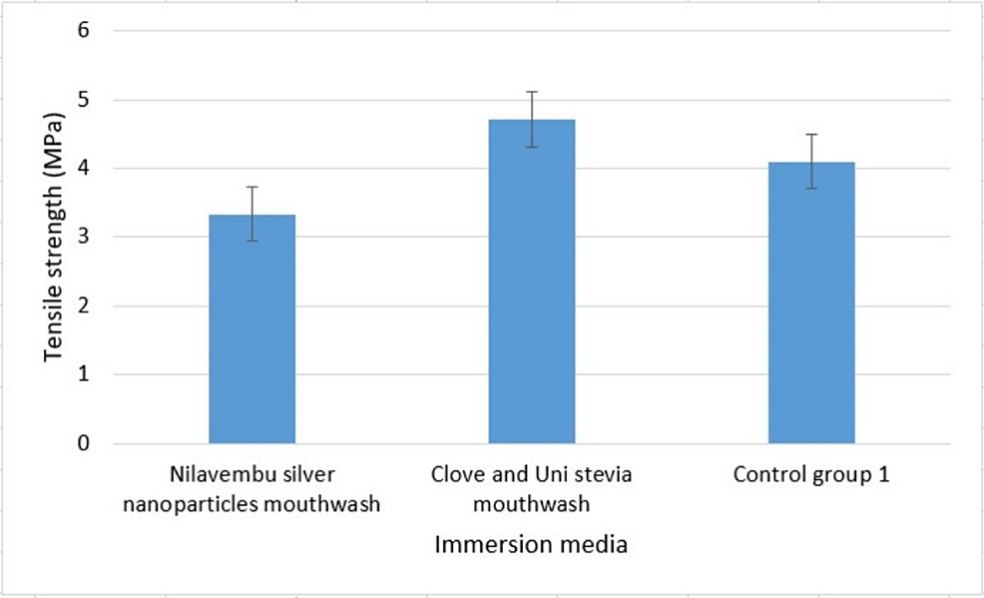
The mean tensile strength of Vicryl sutures after immersion in different media Test group 1 (Vicryl suture immersed in nilavembu silver nanoparticles mouthwash), test group 3 (Vicryl suture immersed in clove-uni stevia mouthwash), and control group 1 (unimmersed Vicryl suture)

**Figure 3:**
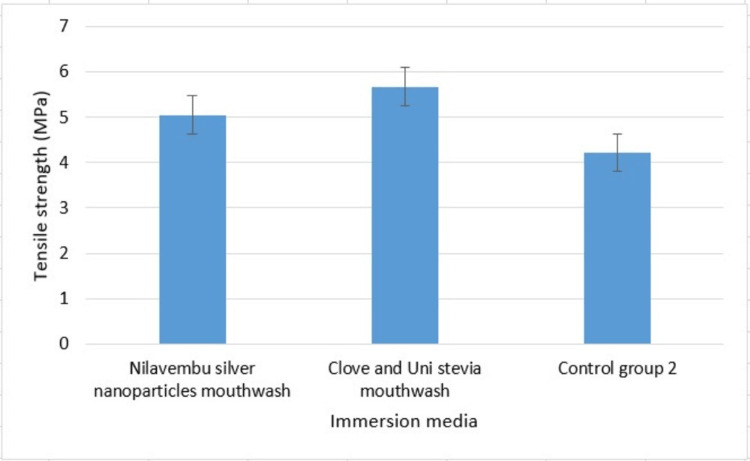
The mean tensile strength of Seamcryl sutures after immersion in different media Test group 2 (Seamcryl sutures immersed in nilavembu silver nanoparticles mouthwash), test group 4 (Seamcryl sutures immersed in clove-uni stevia mouthwash), and control group 2 (unimmersed Seamcryl sutures)

Throughout and after the period of soaking in different media, all the suture materials remained undamaged and showed no signs of visual degradation. While being tested on a universal testing machine, each suture specimen displayed a clear breaking point. Figure 2 shows a bar chart depicting the average tensile strength of Vicryl material after immersion in different media, and Figure 3 shows a bar chart depicting the average tensile strength of Seamcryl material after immersion in different media.

Table 1 shows the comparison of the mean tensile strength of Vicryl after immersion in different media: test group 1 (Vicryl sutures immersed in nilavembu silver nanoparticles mouthwash), test group 3 (Vicryl sutures immersed in clove-uni stevia mouthwash), and control (unimmersed Vicryl sutures).

**Table 1:** The comparison of the mean tensile strength of Vicryl after immersion in different media SD: standard deviation

Groups	Mean	SD	p-value
Test group 1 (Nilavembu silver nanoparticles)	3.3377	0.15166	0.001
Test group 3 (Clove-uni stevia)	4.7133	0.10066	
Control group 1 (Unimmersed)	4.1033	0.12503	

The average tensile strength was shown to be higher in test group 3 (4.7133) than in test group 1 (3.3377) and control (4.1033), with a p-value of 0.001, which is statistically significant (one-way ANOVA test).

Table 2 shows the comparison of the average tensile strength of Seamcryl after immersion in different media: test group 2 (Seamcryl sutures immersed in Nilavembu silver nanoparticles mouthwash), test group 4 (Seamcryl sutures immersed in clove-uni stevia mouthwash), and control (unimmersed Vicryl sutures).

**Table 2:** The comparison of the mean tensile strength of Seamcryl after immersion in different media SD: standard deviation

Groups	Mean	SD	p-value
Test group 2 (Nilavembu silver nanoparticles)	5.0467	0.07122	0.000
Test group 4 (Clove-uni stevia)	5.6667	0.38083	
Control group 2 (unimmersed)	4.2200	0.17349	

The mean tensile strength was considerably higher in test group 4 (5.6667) than in test group 1 (5.0467) and control (4.2200), with a p-value of 0.000, which is statistically significant (one-way ANOVA test).

## Discussion

The current research analysed the effect of nilavembu silver nanoparticles mouthwash and clove-uni stevia mouthwash on the tensile strength properties of Vicryl and Seamcryl polyglactin sutures. The suture material is chosen for different oral surgical procedures based on its properties and versatility. In an experimental investigation, the mechanical stability of surgical suture materials was evaluated under a variety of pH conditions. Various studies looked at how routinely prescribed mouthwashes affected the mechanical characteristics of suture materials used in dental surgery [13]. The synthetic suture material polyglactin 910 (PLG 9110) consists of a mixture of 90% glycoside and 10% L-lactide coated with a uniform layer of calcium stearate. The use of co-polymers improves durability and extends the time that the suture can be left in the oral cavity. Polyglactin has better ligation and knotting capabilities when it comes to soft tissue grafts used in oral surgical procedures for periodontal treatment.

A suture is a medical device that holds body tissues together after an operation or damage. Polymers are used to make the majority of suture materials used globally. Monomers are the small, repetitive components that make polymers. The interactions between polymer chains define the suture's characteristics, which rely on its molecular structure. When a polymer is used to make a suture, its structural qualities, such as molecular weight, level of entanglement, forces acting between molecules, and hydrogen bonding, might affect the polymer's performance, drawability, and strength. An inevitable component of uncomplicated healing is clot formation and its stabilisation in the recovery phase immediately after surgery. In order to accomplish this, sutures are essential. The biomechanical characteristics of suture materials are essential for balancing the physical tensile stresses operating on the edges of the wound [11]. The amount of length change a material experiences prior to the breaking point is referred to as the strain of that material.

The effect of chlorhexidine and Listerine mouthwashes on the tensile strength of absorbable sutures Vicryl and Monocryl were studied to evaluate the influence of these commercially available synthetic mouthwashes on the physical characteristics of the suture strength and stability, and it was found that the tensile strength of Vicryl sutures significantly increased in chlorhexidine and Listerine, and a significant decrease in the tensile strength of all sutures was observed after day 10, regardless of the immersion solution [14].

Vicryl may be suggested for surgical procedures where three to five days of tissue immobilisation are adequate to aid healing. During the first few days following surgery, clove-uni stevia mouthwash can be recommended. Vicryl should be avoided in favour of Seamcryl if a two- to three-week immobilisation is necessary. In medical situations, clove-uni stevia mouthwash can be given. Previous research has suggested that absorbable materials are more resilient to tension than nonabsorbable ones [15]. Polyglactin (Vicryl) sutures were shown to retain their tensile strength up to day 10 but lose most of it by day 14. Vicryl's tensile strength was shown to be lower in saliva than in soy, saline, or milk, according to another study [16]. However, few studies have reported that Vicryl outperforms natural sutures in terms of breaking power. Particularly after soaking in physiological and acidic pH solutions, this is obvious. They added that silk appears to be the non-absorbable suture that is most sensitive to changes in pH [3, 17].

According to a few previous studies, Teflon (PTFE) and polyglactin (Vicryl) sutures maintained their strength after 21 days of testing. Monofilament and polyglactin (Vicryl) demonstrated better tensile strength than black silk (BS) and PTFE [17]. The pH levels have a greater impact on absorbable sutures' performance outcomes than on nonabsorbable ones [18]. It is also suggested that settings that are both acidic and alkaline can hasten the deterioration of absorbable sutures [1,17]. Meanwhile, synthetic absorbable sutures have been shown to degrade faster in alkaline conditions. When comparing the rate of deterioration of various suture materials to that of diverse equine physiological and pathological fluids, it was discovered that multifilament suture materials degrade more quickly than monofilament sutures [19].

The results of this study will help in the selection of appropriate mouthwash for postoperative oral care through evidence-based decision-making. With the help of this knowledge, dental professionals can make sure that the oral hygiene regimen promotes the best possible wound healing and prevents any negative effects on suture integrity. Patients can also profit from understanding which mouthwash options are compatible with polyglactin sutures, enabling them to make educated decisions and actively engage in their dental care throughout the postoperative period.

Limitations

This investigation on the effect of the tensile strength of polyglactin sutures with different herbal mouth rinses, however, has a few limitations. Although simulation of the oral environment with respect to physiological conditions was done, the present in vitro study lacks microbiological and pathological components that could have had an impact on the findings. Other than the effect of the functional elements of mastication, swallowing, and speaking, the experimental setup could not reproduce these processes. The future scope of the study may include studying the effects of various orally applied agents on the mechanical properties of different suture materials used in specific types of tissues.

## Conclusions

Given the constraints of the current investigation, as clove-uni stevia mouthwash was shown to increase the tensile strength of the polyglactin sutures, it can be suggested for oral hygiene maintenance in the post-surgical phase. However, clinical trials are needed further to support a general suggestion for the selection of mouthwashes for certain suture materials in all clinical circumstances. Therefore, while recommending mouthwashes to patients postoperatively, oral surgeons should exercise caution for effective wound closure.
